# Information on COVID-19 and Psychological Distress in a Sample of Non-Health Workers during the Pandemic Period

**DOI:** 10.3390/ijerph17196982

**Published:** 2020-09-24

**Authors:** Carlos Ruiz-Frutos, Mónica Ortega-Moreno, Adriano Dias, João Marcos Bernardes, Juan Jesús García-Iglesias, Juan Gómez-Salgado

**Affiliations:** 1 Department of Sociology, Social Work and Public Health, Faculty of Labour Sciences, University of Huelva, 21007 Huelva, Spain; frutos@uhu.es (C.R.-F.); juanjesus.garcia@dstso.uhu.es (J.J.G.-I.); 2 Safety and Health Postgraduate Programme, Universidad Espíritu Santo, Guayaquil 092301, Ecuador; 3 Department of Economy, University of Huelva, 21007 Huelva, Spain; ortegamo@uhu.es; 4 Public (Collective) Health Grade Program, Botucatu Medical School, São Paulo State University/UNESP, Botucatu 18618-687, Brazil; dias.adriano@unesp.br (A.D.); jmbernardes@yahoo.com (J.M.B.)

**Keywords:** psychological distress, COVID-19, coronavirus, pandemic, knowledge, internet, information, occupational health, non-health workers, mental health

## Abstract

Methods by which the population should be informed when going through a pandemic such as COVID-19 have been questioned because of its influence on the adoption of preventive measures and its effects on mental health. Non-health workers are at risk of psychological distress from exposure to contaminated people or materials or by having to stay at home and adapt their activity to telework. The objective of the study is to analyze information the public receives about COVID-19 and its influence on their level of distress. For this, 1089 questionnaires from non-health workers were collected online between 26 March and 26 April 2020 in Spain, and analysed and distributed by snowball effect. 492 participants carried out essential activities away from home, and 597 did so from home. They were surveyed about information received about COVID-19 regarding its source, time, assessment, or the beliefs expressed in it. Mental health was also measured with Goldberg’s General Health Questionnaire (GHQ-12). The classification and regression tree (CART) method was used to design a binary tree with sample cases. It has been found that the time spent learning about COVID-19 and the level of knowledge about symptoms, pathways, prevention, treatment, or prognosis are associated with the level of distress, where 25% of participants were found to have spent more than 3 h daily on this activity. Social media and television are the most widely used sources, but they are considered to be of lower quality and usefulness than official sources. There is greater confidence in healthcare professionals than in the health system, and the main concern of those working away from home is spreading the virus to family members. It has been concluded that there is a need to enhance quality and truthful information on the Internet for non-health workers due to its accessibility, which should be constantly updated, a fact which international and national public bodies, research centers, and journal publishers have begun to understand during the current pandemic. Such quality information is needed to combat distress.

## 1. Introduction

On 31 December 2019 in Wuhan, Hubei province, China, SARS-CoV-2 generated the pandemic known as COVID-19, which led to the WHO’s declaration of an international public health emergency on 30 January 2020 [1]. This is a pandemic on which bibliographical reviews have been conducted regarding its etiology, pathology or clinical behaviour [2], epidemiology, diagnosis, treatment, and prevention by vaccines [3], or its impact on mental health [4]. The phenomenon is unpredictable in development [5] and, therefore, its enormous effects on the world’s economy are also unpredictable [6]. In Spain, the state of alarm was declared on 14 March 2020 by the Spanish Government and then, on 29 March, measures were strengthened [7]. A declaration was made involving limiting the movement of persons and suspending any face-to-face educational activity, any commercial, cultural, leisure, and sporting activities, worship activities and civil and religious ceremonies including funerals and, in general, any activity involving the concentration of persons. Subsequently, secondary Spanish legislation was established that limited mobility to the maximum, except for activities considered essential.

At the time of writing the present article, early August 2020, the COVID-19 pandemic has produced 18 million contagions worldwide and 700,000 deaths [8], with slightly higher figures on the Johns Hopkins University website [9]. In Spain, 305,000 contagions were counted and 28,499 deaths [8]. The WHO, on its website, provided preventive instructions that focus on: regular hand hygiene with alcohol-based hand sanitizer or soap and water; maintaining safety distance; avoiding crowded places; refraining from touching eyes, nose, and mouth; covering mouth and nose with bent elbow or handkerchief when coughing or sneezing; staying at home and self-isolating when with mild symptoms; and in case of fever, cough, and shortness of breath; seeking medical attention, but doing so in advance by phone so as to be referred to the appropriate health center, as well as following the instructions of the health authorities and staying informed with the latest information from reliable sources such as the WHO [8] or local and national health authorities. Actually, the problem is that Spanish health authorities, like those of most countries and international agencies, have changed their preventive recommendations throughout the pandemic period. As an example, at first the use of a mask was not recommended to the population, and it is not known whether this was due to lack of availability, while in August 2020 it has been established as mandatory in most geographical areas of Spain, both in closed and open spaces, including beaches, even if the safety distance is maintained. After the relative return to normality, and after the first increases in outbreaks and in contaminations, regulations have been issued that once again have closed nightlife venues and prohibit smoking on public roads or in outdoor spaces when a minimum interpersonal distance of at least 2 m cannot be respected [10].

A greater personal sense of danger caused by the COVID-19 pandemic tended to increase anxiety levels, while greater individual resilience tended to decrease the rate of this apprehension. However, higher education was related to higher levels of depression and anxiety [11]. Emerging adults (aged 18–29) practiced significantly less social distancing compared to adults and the elderly and were more likely to leave home for non-essential reasons. A possible explanation for these behaviors may be linked to this group reporting the lowest anxiety levels compared to adults and the elderly [12]. Information/guidelines have been issued regarding implementation of innovative infection-control methods including personal protective equipment (PPE), which might provide a sense of safety, and in turn might reduce psychological distress and subjective overload [13].

According to the Dictionary of the American Psychological Association, psychological distress is a set of painful mental and physical symptoms that are associated with normal fluctuations of mood in most people. In some cases, however, psychological distress may indicate the beginning of major depressive disorder, anxiety disorder, schizophrenia, somatization disorder, or a variety of other clinical conditions. It is deemed to be assessed by many putative self-report measures of depression and anxiety [14].

Changes in preventive recommendations by health authorities and lack of consensus in the messages issued by media sources have raised public concern and bewilderment. We know that, by providing adequate information on social and other media, citizens’ engagement increases, and so does compliance with COVID-19 preventive measures [15]. In previous epidemics, we have seen that the Internet is a good way to transmit such empowering information to the population [16]. Instead, perceiving that adequate protective measures are not available has led to mental disturbances and post-traumatic stress [17], to which the direct effects of the pandemic must be added: stress, depression, and anxiety [18].

It may be debated whether spending more or less time receiving information about COVID-19 increases or reduces stress or concern [19,20], but it seems clear that believing that measures are effective in preventing contagion facilitates compliance [21,22,23]. From other pandemics we have learned that one of the factors that most contributed to reducing the psychological impact of having to be isolated at home is having received clear and consistent information [24,25]; this also reduces the perception of risk [16].

Some studies have found the Internet more reliable than traditional channels [16], although how counterfeit information is disseminated over the Internet [26], as has been seen in previous epidemics [27,28], has been widely analyzed. What is undoubted is that today, especially in such ever-changing situations during this pandemic, the Internet is seen as the ideal means for people to receive up-to-date information, regardless of whether the source of information is official or not. However, multiple studies have found a link between medical conspiracy beliefs and reluctance to engage in health-protective behaviors with regard to vaccination [29]. YouTube and Facebook have been identified as major vectors for dissemination of misinformation [30]. COVID-19 conspiracy beliefs have been defined as beliefs that the COVID-19 public health crisis was produced through intentional agency (whether through manufacture of the coronavirus or through deliberate exaggeration or incorrect attribution of negative health outcomes) [31]. Specific health protection behaviors against COVID-19 have been analysed, and it has been concluded that, when used as a source of information, unregulated social media may present a health risk that is partly, but not wholly, reducible to their role as disseminators of health-related conspiracy beliefs [31].

Therefore, promotion of the use of official public health agencies’ websites to offer information the preventive measures against COVID-19 on the Internet has been urged, so that users have high quality and up-to-date information [32]. This is something that public bodies have already learned, creating specific websites that provide up-to-date information, e.g., the World Health Organization [8]; the International Labor Organization [33]; universities with prestigious research centers [9]; and health authorities [34]. Magazine publishers, especially those with open access, have set up websites or created specific webinars to disseminate published information about COVID-19 [35]. Even some editors of pay journals have offered information for free, temporarily during the pandemic period [36,37].

The objective of this study is to know whether the information received during the pandemic period, when analyzing sources of information, time dedicated to it, knowledge or beliefs, has influenced the level of psychological distress in a sample of non-health workers in Spain.

## 2. Materials and Methods

### 2.1. Design

This is an observational cross-sectional descriptive study.

### 2.2. Measuring Instruments

For data collection, an ad hoc questionnaire was developed. Prior to the design of this questionnaire, a literature review was conducted. Studies published on previous pandemics and epidemics were reviewed focusing on information provided to the population. Variables and items related to the purpose of this study were extracted. The research team designed a pilot version of the questionnaire which was assessed by an expert panel formed by psychologists, doctors and nurses. When content validity was obtained, the reviewed version was tested among 57 participants chosen by convenience. No issues in understanding or answering the questionnaire were remarked. 

Goldberg’s General Health Questionnaire (GHQ-12) [38] was used to assess mental health and psychological adjustment, which serves as a screening for non-psychotic psychiatric disorders and has previously been used in other epidemics such as SARS [39]. This is a scale of 12 items, with four response options and an overall rating from 0 to 12. A Cronbach’s alpha of 0.861 was obtained, and a cut-off point of 3 was established. Specific questions about the COVID-19 pandemic were added, such as the type of information source used and the amount of time this was consulted, or the number of hours per day listening to, reading, or watching pandemic-related news, how the degree of veracity of the information received was assessed and whether it contrasted with official sources (dichotomic response YES/NO). Participants were also asked to evaluate from 1 to 10 their level of concern about COVID-19 (being infected or transmitting the virus to their close family members), their confidence in health professionals and in the health system, their perception of the risk of getting infected, the health consequences of the infection, and the difficulty of obtaining treatment. They assessed from 1 to 10 their degree of information regarding COVID-19 symptoms, prognosis, treatment, transmission routes, preventive measures, and information provided by their company. The accessibility, quality, quantity, and usefulness of the information received was assessed through a Likert-type scale that ranged from 1 to 5, in which 1 = very low, 2 = low, 3 = intermediate, 4 = high, and 5 = very high. One item assessed information on symptoms, prognosis, treatments, transmission pathways, and preventive measures, using a five-choice Likert-type response.

### 2.3. Procedure and Sample

The questionnaire was distributed online through the online survey platform Qualtrics^®^ and sent to the email lists of professional groups who were invited to participate. Participants were invited to disseminate the questionnaire among their work colleagues to trigger a snowball effect. Participants completed the survey using different electronic devices (tablets, PCs, and mobile phones) with internet access. Data collection took place between 26 March, 13 days after the start of the health alarm state, and April 26.

Of the sample of 1089 non-health workers, 492 performed essential activities and worked away from home during the health alarm period, and the remaining 597 workers carried out their work from home. Inclusion criteria were being 18 years of age or older, a currently active worker, and having accepted informed consent. Exclusion criteria were being under 18 years of age, being a healthcare worker, and not residing in Spain at the time of answering the questionnaire.

Among the participants forming the sample, 66% (719) were women, and the average age was 42.10 years (SD = 11.09 years). 45% of respondents belonged to the essential activities group, and therefore worked away from home during the pandemic. 10.5% were self-employed, 37.5% public workers, and the rest private company workers; 62.4% were married or living with a partner, 27.7% were single, and the remaining 9.8% were separated, divorced, or widowed. In addition, 54.6% had children under the age of 16.

### 2.4. Ethical Considerations

Participants consented to voluntarily participate after being informed about the purpose and procedure of the study. Participation in the study did not involve any risk or benefit for the participants. Data were collected and recorded anonymously, maintaining at all times the confidentiality of the information. The study was approved by the Research Ethics Committee of Huelva, of the Andalusian Health Administration (PI 036/20).

### 2.5. Data Analysis

The frequencies, percentages, position, and dispersion measures, depending on the type of variable, allowed us to present a descriptive analysis of the data. Then the Student’s *t*-test for independent samples and the chi-squared association test were used to contrast differences and/or discover non-existent relationships between the different variables, related to beliefs about the outbreak and about information provided on the pandemic, and also regarding the presence or not of psychological distress. The complete sample was categorized as non-health workers and those who were working away or from home.

The classification and regression trees (CART) method was used to design a binary tree with sample cases. Optimal cut-off points were selected to improve overall purity by minimizing the adjustment statistical value. Thus, cases in each part were similar within that part, and different from cases of any other part. Terminal nodes showed the predominant class, the proportion of psychological distress cases within the node, and the percentage of node cases over the sample total. The model allows the prediction of the percentage of those suffering psychological distress in new cases.

The analyses were carried out with the statistical software SPSS 26.0 (SPSS Inc., Chicago, IL, USA) and R version 4.0.0 (IBM, Armonk, NY, USA).

## 3. Results

### 3.1. Sociodemographic Data and Psychological Distress

Among the 1089 respondents, 65.1% (709) showed psychological distress. When analyzing the sample by gender, 71.6% of women and 52.4% of men showed psychological distress. In addition, it is possible to observe a statistically significant association between gender and distress (*p* < 0.001). By age, the percentage of cases with psychological distress was higher (69.4%) among the younger (under-median age, 43 years), than among the older (60.4%), and there was a statistically significant association between variables (*p* = 0.002). The association was also statistically significant between the type of home, with (75.2%) or without (24.8%) outdoor space, and distress (*p* = 0.025).

Having or not having psychological distress did not show a significant association with other sociodemographic variables: workplace during the pandemic (*p* = 0.172), type of work (*p* = 0.062), marital status (*p* = 0.363), or having responsibility for children under the age of 16 *(p* = 0.346).

### 3.2. Beliefs about the Outbreak and Psychological Distress among Non-Health Workers

There is a clear association between most concerns about COVID-19 and the presence of psychological distress (Table 1). Thus, in a range of 1 to 10, concern about COVID-19 among non-health workers is M = 8.17 (SD = 1.73), being higher among those with psychological distress, both in the group of workers who carried out their activity away from home and those who did so from home. The highest value is found in “The degree of concern to be a carrier and transmit the virus to family members, close persons, or patients”, with M = 9.01 (SD = 1.70). Also exceeding the value of 8 is “The probability of surviving COVID-19 if infected now or getting infected” and “Confidence in the ability of healthcare professionals to diagnose or recognize COVID-19”. On the other hand, those categories that obtain lower values are “The risk of being infected with COVID-19”, with M = 5.39 (SD = 2.38) and “Health consequences of the infection”, with M = 5.92 (SD = 2.39).

There are statistically significant differences between workers working away from home or doing so from home in the variables: “Risk of being infected by COVID-19” (t = −4.475, *p* < 0.001) and “Degree of concern to be a carrier and transmit the virus to family members, close persons, or patients” (t = −3.249, *p* = 0.001).

### 3.3. Accessibility, Quantity, Quality, and Usefulness of Media and Official Information Sources on COVID-19, and Level of Psychological Distress

Table 2 shows that 74.56% of participants claim to have high or very high accessibility to the media, which drops to 48.21% compared to access to official information sources. 85.22% state that the amount of information received from the media is high or very high, a percentage that drops to 49.13% compared to that from official information sources. The quality of the information received by the media is considered high or very high by 16.72% of participants, and in relation to the official information sources this rises to 28.1%. As for the usefulness of the information received, 24.98% believe that media information is of high or very high usefulness, while this assessment for official information sources rises to 32.78%. We have found an association between the degree of accessibility, quantity, quality and usefulness of information and the means of obtaining said information by different communication or official channels (*p* < 0.001 in the four cases).

### 3.4. Sources of Information, Time Spent Receiving Information, and Psychological Distress

62.4% of non-health workers stated that they consulted more than two sources of information about the pandemic, and 45.5% four or more. The mean number of daily consulted sources was 3.37 (SD = 1.98), but there is no statistically significant difference between workers who carry out their activity away from home and those who work from home (t = 1.831; *p* = 0.067).

98.1% of non-health workers spent at least an hour a day watching, reading, or listening to news related to COVID-19; 58.4% did so between one and two hours a day; and 25.3% spent more than 3 h a day. The mean was 2.84 (SD = 2.53) hours per day. This time was higher among workers with psychological distress (GHQ > 3, *p* < 0.001), but this difference was not found in the subgroup of workers involved in essential activities who carried out their work away from home (*p* = 0.129).

No differences were found in the number of hours consulting information among those who carried out their work from home and those who did so away from home (t = −1.513; *p* = 0.130).

The most consulted type of information was on “symptoms”, M = 7.05 (SD = 2.35), followed by “preventive measures”, M = 6.99 (SD = 2.47), and “transmission pathways”, M = 6.82 (SD = 2.45). Workers gave a high score to the information received by their companies, M = 6.16 (SD = 2.89) (Table 3).

**Figure 1:**
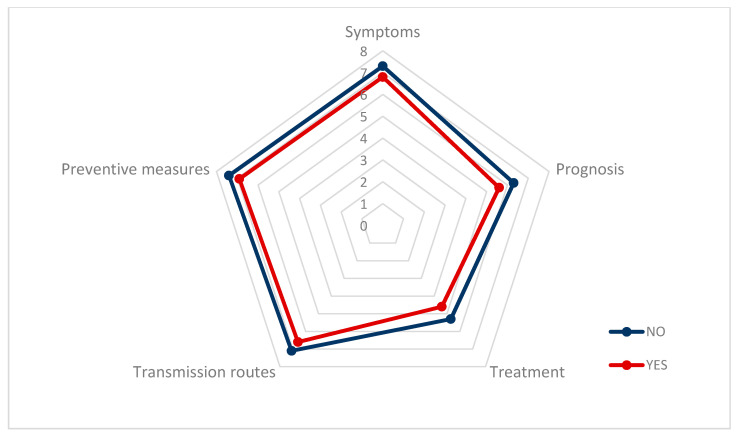
Information that non-health workers believe about COVID-19, distinguishing between the presence or absence of psychological distress.

Figure 1 shows how the information non-health workers have about COVID-19 regarding symptoms, preventive measures, transmission pathways, treatment, and prognosis is lower, in all of the variables, among workers with psychological distress than among workers with lower values of distress.

### 3.5. Media or Platforms to Receive Information about the COVID-19 Pandemic

The most commonly used means of receiving information about COVID-19 were social media (77.41%), followed by television (63.82%) and friends or family (42.33%). Official media, such as the websites of official agencies or scientific societies, appear in fourth place (41.05%) (Table 4).

### 3.6. Classification and Regression Tree for the Level of Psychological Distress Based on Beliefs Regarding the Pandemic

**Figure 2:**
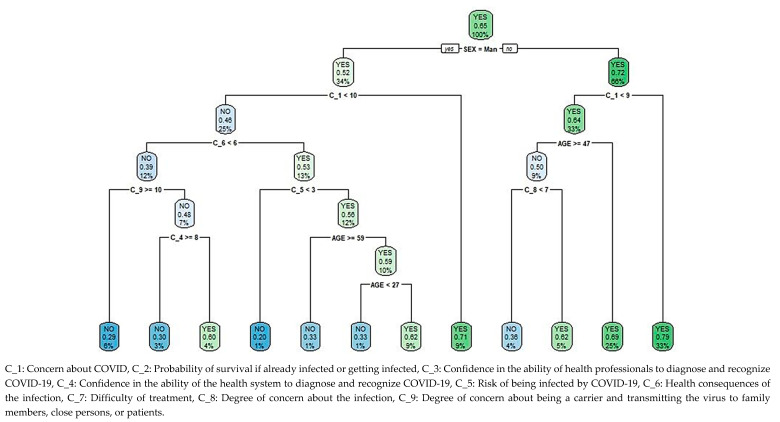
Classification and regression tree (CART) for psychological distress among non-health workers during the pandemic, based on beliefs about the outbreak.

The classification and regression tree (CART) for the level of psychological distress depending on beliefs about COVID-19, Figure 2, shows 1089 cases in the root node, of which 65.11% (1051) have psychological distress. A second node differentiates by gender, collecting a psychological distress rate of 52.43% (194 cases) in 33.98% (370) of men, which increased to 71.63% (515) among women. For both genders, the most significant variable on a third level is concern about COVID-19, obtaining different criteria for the construction of the tree.

For 8.72% (95) of men with a high level of concern about COVID-19, that is a cut-off point greater than or equal to 9.5 out of 10, the percentage of psychological distress is 70.53% (67), only surpassed by women who rate their level of concern with values greater than or equal to 8.5 out of 10 (32.78% of the population, 357 cases), with 79.27% (283) of cases showing psychological distress. In women with lower levels of concern, this score is below 8.5, the percentage of cases with distress is 69.03% in those under 46.5 years of age (24.61% of the total population, 268 cases), and depending on whether the degree of concern about becoming infected is high (greater than or equal to 6.5 out of 10), the percentage of cases is 62% (31), decreasing to 36.36% (16) otherwise. This last level has the lowest number of female cases, 4.04% (44), and is the only one in which the percentage of cases without psychological distress exceeds that of cases with distress.

25.25% (275 cases) in the male population with a lower level of concern about COVID-19, i.e., below 9.5, ranked according to a fourth level, which considers the health consequences of the infection. For the 12.49% (136) who consider there to be fewer health consequences, i.e., a score less than 5.5 out of 10, the percentage of cases without distress is approximately 70%, regardless of the degree of concern about being a carrier and transmitting the virus to family members, close persons, or patients. There is an exception though in 3.94% (43) of the population, whose degree of concern about being a carrier is lower and confidence in the health system’s ability to diagnose or recognize COVID-19 is scored lower than 7.5, for which group the percentage of cases with distress is 60.74% (26). The highest percentage of cases without distress, 80% (8 cases) among males with the lowest levels of concern about COVID-19, corresponds to 0.92% (10) of the population who, despite considering greater health consequences, i.e., a score above or equal to 5.5, consider that they have a low level of risk of being infected, i.e., a score lower than 2.5 out of 10. Otherwise, i.e., with a score for risk of infection equal to or greater than 2.5, the percentage of cases with distress is 61.76% for non-health workers aged between 26.5 and 58.5 (1.10% of the population, 12 cases); outside this range, the percentage of cases with psychological distress drops to 33.33% in 10.74% (117) of the population.

## 4. Discussion

Pandemics are known among health professionals to generate anxiety, depressive disorders, or post-traumatic stress [40], and possible behavioral responses to massive threats have been proposed [41]. However, the effects on other workers in essential activities that have also been exposed to people who may be infected have been less studied. We have found that in this group, COVID-19 is a concern that translates into an extensive search for information that can answer their questions.

In our study, a clear association between the level of information received on COVID-19 and the level of psychological distress has been observed. This is consistent with previous studies where the importance of quality information is associated with the level of psychological distress [24,25].

Official information sources and scientific societies rank fourth among the means through which the analysed population received information. This confirms what had already been learned from previous epidemics, i.e., the importance of improving information on social media and the Internet [16], as the Internet is the most widely used means of learning about COVID-19 [42]. It is understandable that many try to avoid the stress produced by lack of information by seeking the answer to their concerns on the Internet [43], as it is a widely used means to resolve health-related questions [44].

If the accessibility and amount of information received via the media is greater than that from official sources, then information from these official sources is perceived as of higher quality: 28.1% consider it high, a percentage that drops to 16.72% for information received through the media. The usefulness of this information is also considered greater if it comes from official media sources (32.78%) rather than from the rest of the media (24.98%). This is consistent with previous studies that have detected low quality of information on COVID-19 on the Internet [32,44]. However, it is essential for official and quality information to be available on the Internet and updated [16,45], as has been admitted during the current pandemic by international organizations [8,33], scientific entities, [9] and journals’ editorial boards [35,36,37]. The need to increase the visibility of official information seems evident because, although it is well valued, it does not seem to reach the working population. This requires that official bodies make a greater effort to disseminate information by employing the same attractive tools used by communication network platforms.

It is difficult to assess the quality of the information distributed by social networks, the most commonly used sources in the present study (77.41%), because of the difficulty in discriminating fake news. COVID-19-related content was identified on social media as follows: (a) updating new cases and their impact; (b) frontline reports on the epidemic and its prevention measures; (c) experts’ opinions on outbreaks of the infection; (d) frontline health services; and (e) global scope of the epidemic and identification of suspected cases [46]. The topics of greatest interest have also been identified: origin and transmission routes; magnitude of the impact on countries (infected and deaths, stress and fear, economy, movement restrictions), means of spreading, and risk control [47].

It is known that there are mechanisms that facilitate the dissemination of bogus information, that generate false beliefs in individuals that, once assimilated, are difficult to correct. It is known that social homogeneity is the main driver of dissemination of content, and usually starts from a friend with the same profile [32], to which we must add the high number of articles published in such a short time [48], enhanced by predatory magazines [48,49]. Broadcast and print media are regulated, while social media are not. This means that misinformed and incorrect news, such as conspiracy beliefs, can be sustained at length on social media platforms [30,31].

Our study shows that there is great confidence in the ability to diagnose COVID-19 disease by health professionals (M = 8.28, SD = 1.84), yet this level of confidence is lower regarding the ability of the public health system to diagnose the disease (M = 6.96, SD = 2.25). This lack of confidence in the health system but not in its professionals can be explained by the recognition of health professionals who have had to risk their lives without adequate means of protection, and of lack of diagnostic tests or resources for treatment, triggered by inadequate provision of the public health system for such public emergencies. After the frequent changes in the recommendations on preventive measures to be taken by the population, public bodies, the WHO and health authorities have been able to state that the current epidemic may have been more unpredictable than previous ones. This may have unleashed an increase in the emergence of myths and erroneous news, which in turn generates greater concern among the population [50].

The sample was less concerned with becoming infected than with the possibility of infecting others (relatives), as was also seen in previous studies [51]. This is something that will logically take place among non-health workers in essential activities who carry out their activity away from home, and not among those who are confined at home, because those who work away from home are the most exposed to being infected.

One in four workers devoted more than three hours a day to learning about COVID-19, and those with psychological distress spent even more time. In previous studies, the most unknown risks are found to be more threatening [52], while others have shown that less health knowledge is associated with less concern about becoming infected [53]. The use of preventive measures has also been associated with the level of information [45,54]; more information is associated with perceiving a higher risk and with greater compliance with preventive measures [22,23]. Workers who are working from home do not consult more information than those who are working away from home during the pandemic, which is unexpected because many have had to adapt their activity to telework and, as a result, they have had greater access to the Internet. The distress generated by the increase in time spent receiving information on COVID-19 can be countered by that originating in external workers being exposed to infected persons or objects [55], or the stress associated with confinement at home [56].

We see how, in other previous studies, women have a higher percentage of psychological distress [56,57,58,59,60], influenced by their level of concern about COVID-19. This may have been caused by a higher percentage of female responses to the questionnaire. Men’s level of distress will overcome that of women only if they present a much higher level of concern about COVID-19 than women. So, the level of distress among men with a level of concern equal to or greater than 9.5 out of 10 is surpassed by that of women concerned about COVID-19 in a level equal to or greater than 8.5 out of 10. Among men with less concern about COVID-19, the level of distress is associated with perception of the health consequences, in particular by the possibility of infecting family members, as in previous studies [51], and with the level of confidence in the health system.

Male non-health workers with a low level of concern about COVID-19 have a low level of distress, even if they consider the possibility of greater consequences for their health, if they perceive a low risk of being infected. This outcome could lead us to interpret the perception of the risk of getting infected as one of the main variables that conditions psychological distress, which is in line with other studies [22]. In this group, a high perception of risk and an age between 26.5 and 58.5 years generates greater distress than among non-health workers outside this age range. Thus, age is another variable that intervenes in the generation of distress, as has also been found in previous studies [61].

From our study data, we can infer that non-health workers who carry out essential activities away from home and have therefore been exposed to getting infected by contact with contaminated people or objects should be able to receive truthful information about COVID-19, for which the public bodies’ websites [32] have been proposed. Occupational Health and Safety physicians at companies may be another good option. In light of all this, it is highly advisable to implement active measures that complement the information made available by official media together with the use of counsellors who can provide psychological support via telephone that can mitigate the psycho-emotional impact generated by COVID-19 [62].

The data collection at the beginning of the health alarm, at which point the biggest slope in the contagion curve was taking place, is one of the values of this study, but there are some limitations. A random sample could not be made and there is a higher percentage of women, but this was partly offset by questionnaires from all provinces of Spain and by the high number of questionnaires. There are differences in the measures taken by each country, which range from the type of movement restriction or confinement at home, and other preventive measures to be taken, making it difficult to compare the results at the international level. Further questionnaires are planned to analyze data at different stages of the pandemic, linking this with measures that have been taken to deconfine or to return to the so-called “new normal” and the response to the occurrence of new outbreaks.

## 5. Conclusions

In the present study, one in four participants of the population analysed, i.e., non-health workers, devoted more than 3 h a day to learning about COVID-19, with no differences between workers who carried out their activity away from home and those who did so from home, and this has greatly influenced their level of psychological distress. Although the most widely used means of receiving information are social networks and, secondly, television in terms of accessibility and quantity, such information is considered to be of lower quality and usefulness than information from official sources. Social media can be a vehicle for false news, although there are mechanisms to identify this, and being the main source of information social media must be a priority target of analysis in to counter inaccurate reports.

Less knowledge about the symptoms, pathways of transmission, prevention, treatment, and prognosis of COVID-19 is associated with low values of psychological distress.

Greater confidence has been shown as regards the ability of health professionals to diagnose the disease than regarding the capacity of the health system, a result which may be motivated by changes in the information provided by health authorities over the pandemic period. The main concern of workers in essential activities, who were working away from home during confinement, was the possibility of infecting family members upon returning to their homes.

Among the variables found to be associated with distress are sex (higher levels of distress among women), age, concern about COVID-19, perception of health consequences, risk of being infected, possibility of infecting family members, and level of confidence in the health system.

There is a need to improve the quality and truthfulness of information received by non-health workers engaged in essential activities during the COVID-19 pandemic, fostering its online access, given accessibility, so that it is permanently up to date. Eventually, this is something that international and national public bodies, research centers, and magazine publishers seem to have begun to understand during the current pandemic.

## Figures and Tables

**Table 1:** Relationship between variables related to beliefs about the outbreak and psychological distress among non-health workers working during the pandemic.

	Non-Health Workers (*n* = 1089)					Non-Health Workers Working Away from Home (*n* = 492)					Non-Health Workers Working from Home (*n* = 597)				
		GHQ12					GHQ12					GHQ12			
	M (SD)	NO (*n* = 380)	YES (*n* = 709)	Statistics	*p*	M (SD)	NO (*n* = 161)	YES (*n* = 331)	Statistics	*p*	M (SD)	NO	YES	Statistics	*p*
Concern about COVID (C_1)	8.17 (1.73)	7.70 (1.87)	8.42 (1.60)	6.696	<0.001 **	8.17 (1.76)	7.83 (1.79)	8,34 (1.72)	−3.072	0.002 **	8.17 (1.71)	7.61 (1.92)	8.49 (1.49)	−5.901	<0.001 **
Probability in surviving if already infected or getting infected (C_2)	8.10 (1.84)	8.34 (1.78)	7.97 (1.86)	−3.137	0.002 **	8.03 (1.82)	8.36 (1.73)	7.87 (1.84)	2.828	0.005 **	8.16 (1.85)	8.32 (1.82)	8.07 (1.87)	1.642	0.101
Confidence in the ability of health professionals to diagnose and recognize COVID-19 (C_3)	8.28 (1.84)	8.44 (1.78)	8.20 (1.87)	−2.047	0.041 *	8.28 (1.89)	8.47 (1.81)	8.18 (1.92)	1.606	0.109	8.29 (1.80)	8.42 (1.76)	8.22 (1.83)	1.297	0.195
Confidence in the ability of the health system to diagnose and recognize COVID-19 (C_4)	6.96 (2.25)	7.26 (2.18)	6.80 (2.27)	−3.263	0.001 **	6.82 (2.33)	7.17 (2.22)	6.64 (2.36)	2.398	0.017 *	7.08 (2.18)	7.32 (2.15)	6.93 (2.19)	2.126	0.034 *
Risk of being infected by COVID-19 (C_5)	5.39 (2.38)	5.06 (2.42)	5.57 (2.35)	3.387	0.001 **	5.75 (2.37)	5.35 (2.42)	5.94 (2.32)	−2.617	0.009 **	5.10 (2.36)	4.85 (2.39)	5.25 (2.33)	−1.998	0.046 *
Health consequences of the infection (C_6)	5.92 (2.39)	5.71 (2.44)	6.03 (2.36)	2.115	0.035 *	5.93 (2.39)	5.73 (2.39)	6.03 (2.39)	−1.308	0.192	5.91 (2.39)	5.70 (2.47)	6.03 (2.33)	−1.657	0.098
Difficulty of treatment (C_7)	6.89 (2.08)	6.69 (2.14)	7.00 (2.03)	2.295	0.022 *	6.98 (2.11)	6.87 (2.12)	7.03 (2.11)	−0.791	0.429	6.82 (2.05)	6.57 (2.16)	6.97 (1.97)	−2.266	0.024 *
Degree of concern about the infection (C_8)	7.33 (2.37)	6.83 (2.40)	7.60 (2.31)	5.113	<0.001 **	7.47 (2.40)	7.21 (2.25)	7.59 (2.46)	−1.706	0.089	7.22 (2.35)	6.56 (2.48)	7.60 (2.18)	−5.182	<0.001 **
Degree of concern about being a carrier and transmitting the virus to family members, close persons, or patients (C_9)	9.01 (1.70)	8.83 (1.79)	9.10 (1.64)	2.45	0.015 *	9.19 (1.46)	9.27 (1.20)	9.15 (1.58)	0.846	0.398	8.86 (1.85)	8.51 (2.06)	9.06 (1.68)	−3.347	0.001 **

GHQ12 answers (YES/NO) score ranges from 1 to 10; * *p* < 0.05; ** *p* < 0.01.

**Table 2:** Association between information offered about the pandemic and psychological distress among non-health workers working during the pandemic.

		MEDIA					OFFICIAL INFORMATION						
			% GHQ12					% GHQ12					
		*n* (%)	NO	YES	χ^2^	*p*	*n* (%)	NO	YES	χ^2^	*p*	χ^2^	*p*
			(*n* = 380)	(*n* = 709)				(*n* = 380)	(*n* = 709)				
Accessibility	Very low	25 (2.30)	3.16	1.83	7.065	0.132	56 (5.14)	3.68	5.92	5.953	0.203	442.394	<0.001 **
	Low	54 (4.96)	3.42	5.78			128 (11.75)	11.32	11.99				
	Intermediate	198 (18.18)	20.00	17.21			380 (34.89)	32.37	36.25				
	High	438 (40.22)	41.58	39.49			382 (35.08)	38.42	33.29				
	Very high	374 (34.34)	31.84	35.68			143 (13.13)	14.21	12.55				
Quantity	Very low	15 (1.38)	2.11	0.99	4.066	0.397	35 (3.21)	2.89	3.39	7.530	0.110	305.737	<0.001 **
	Low	29 (2.66)	2.89	2.54			123 (11.29)	10.53	11.71				
	Intermediate	117 (10.74)	10.26	11.00			396 (36.36)	31.84	38.79				
	High	304 (27.92)	30.00	26.80			384 (35.26)	39.47	33.00				
	Very high	624 (57.30)	54.74	58.67			151 (13.87)	15.26	13.12				
Quality	Very low	110 (10.10)	9.74	10.30	2.606	0.626	135 (12.40)	11.32	12.98	5.074	0.280	451.026	<0.001 **
	Low	254 (23.32)	21.05	24.54			220 (20.20)	18.68	21.02				
	Intermediate	543 (49.86)	50.79	49.37			428 (39.30)	39.47	39.21				
	High	162 (14.88)	16.58	13.96			263 (24.15)	25.00	23.70				
	Very high	20 (1.84)	1.84	1.83			43 (3.95)	5.53	3.10				
Usefulness	Very low	75 (6.89)	7.11	6.77	6.156	0.188	108 (9.92)	8.42	10.72	6.945	0.139	541.742	<0.001 **
	Low	210 (19.28)	16.05	21.02			196 (18.00)	17.11	18.48				
	Intermediate	532 (48.85)	48.68	48.94			428 (39.30)	37.11	40.48				
	High	246 (22.59)	25.00	21.30			290 (26.63)	29.47	25.11				
	Very high	26 (2.39)	3.16	1.97			67 (6.15)	7.89	5.22				

** *p* < 0.01.

**Table 3:** Sources of information, and time devoted to them, about the pandemic and psychological distress among non-health workers working during the pandemic.

	Non-Health Workers (*n* = 1089)					Non-Health Workers Working Away from Home (*n* = 492)					Non-Health Workers Working from Home (*n* = 597)				
		GHQ12					GHQ12					GHQ12			
	M (SD)	NO (*n* = 380)	YES (*n* = 709)	Statistics	*p*	M (SD)	NO (*n* = 161)	YES (*n* = 331)	Statistics	*p*	M (SD)	NO (*n* = 219)	YES (*n* = 378)	Statistics	*p*
No of sources	3.37 (1.98)	3.29 (1.94)	3.41 (2.00)	0.994	0.321	3.25 (1.98)	3.29 (1.93)	3.23 (2.00)	−0.328	0.743	3.47 (1.98)	3.29 (1.96)	3.58 (1.99)	1.718	0.086
*n*. hours daily	2.84 (2.53)	2.48 (1.98)	3.04 (2.76)	3.861	<0.001	2.97 (2.50)	2.73 (2.17)	3.09 (2.64)	1.519	0.129	2.74 (2.55)	2.30 (1.82)	2.99 (2.86)	3.637	<0.001
Information on COVID-19 *															
Symptoms	7.05 (2.35)	7.36 (2.30)	6.88 (2.37)	−3.241	0.001	6.83 (2.43)	6.99 (2.44)	6.75 (2.43)	−1.033	0.302	7.23 (2.27)	7.64 (2.16)	7.00 (2.31)	−3.353	0.001
Prognosis	5.79 (2.38)	6.30 (2.37)	5.52 (2.34)	−5.173	<0.001	5.57 (2.38)	5.94 (2.38)	5.39 (2.37)	−2.422	0.016	5.98 (2.36)	6.56 (2.33)	5.64 (2.31)	−4.652	<0.001
Treatment	4.79 (2.45)	5.28 (2.58)	4.52 (2.33)	−4.753	<0.001	4.57 (2.41)	5.00 (2.49)	4.36 (2.35)	−2.781	0.006	4.96 (2.46)	5.48 (2.63)	4.66 (2.31)	−3.809	<0.001
Transmission routes	6.82 (2.45)	7.17 (2.35)	6.63 (2.48)	−3.430	0.001	6.63 (2.54)	6.81 (2.52)	6.54 (2.55)	−1.117	0.264	6.98 (2.36)	7.42 (2.19)	6.72 (2.41)	−3.570	<0.001
Preventive measures	6.99 (2.47)	7.36 (2.30)	6.79 (2.54)	−3.731	<0.001	6.75 (2.60)	7.12 (2.38	6.56 (2.69)	−2.317	0.021	7.19 (2.35)	7.53 (2.24)	6.99 (2.39)	−2.749	0.006
Clear and precise information offered by department, service, unit, company *	6.16 (2.89)	6.54 (2.76)	5.95 (2.94)	−3.274	0.001	5.87 (2.99)	6.11 (2.79)	5.75 (3.08)	−1.290	0.209	6.39 (2.79)	6.85 (2.71)	6.12 (2.80)	−3.106	0.002

* Scoring ranges from 1 to 10.

**Table 4:** Means or platforms through which information on the COVID-19 pandemic is made accessible for non-health workers.

Means or Platforms through Which Information on the COVID-19 Pandemic is Made Accessible	No of Cases	Percentage
Social media (WhatsApp, Facebook, Instagram, etc.)	843	77.41
Television	695	63.82
Friends or relatives	461	42.33
Webpages of official organisms or scientific societies	447	41.05
Papers (online or paper)	436	40.04
Google or other search engines	309	28.37
Radio	297	27.27
Mobile phones or apps for official information	131	12.03
Other (professional bodies, companies, …)	52	4.78
